# The Polish version of the Emotion Regulation Questionnaire-Short Form (ERQ-S): Psychometric properties, Polish norms and relationships with psychopathology and well-being

**DOI:** 10.1017/gmh.2024.97

**Published:** 2024-10-28

**Authors:** Paweł Larionow, Karolina Mudło-Głagolska, David A. Preece

**Affiliations:** 1 Faculty of Psychology, Kazimierz Wielki University, Bydgoszcz, Poland; 2 Faculty of Health Sciences, School of Population Health, Curtin University, Perth, WA, Australia; 3 School of Psychological Science, The University of Western Australia, Perth, WA, Australia

**Keywords:** anxiety, cognitive reappraisal, depression, emotion regulation, expressive suppression, norms, psychometric properties, psychopathology, questionnaire, well-being

## Abstract

The Emotion Regulation Questionnaire-Short Form (ERQ-S) is a brief 6-item self-report measure of two emotion regulation strategies, cognitive reappraisal and expressive suppression. It is a short form of the most widely used emotion regulation measure in the field, but currently there are limited data on the performance of the ERQ-S. The aim of this study was to introduce a Polish version of the ERQ-S, examine its psychometric properties and provide Polish norms to aid score interpretation. Our sample was 574 Polish-speaking adults aged 18–69 from the general community in Poland. We examined the ERQ-S’s factor structure and measurement invariance with confirmatory factor analysis. We assessed the concurrent validity of the questionnaire via relationships with psychopathology symptoms and well-being. As expected, the Polish version of the ERQ-S demonstrated strong factorial validity with a theoretically congruent 2-factor structure (cognitive reappraisal and expressive suppression factors), which was invariant across gender, age and education categories. The ERQ-S’s concurrent validity and internal consistency reliability were good. As expected, cognitive reappraisal was significantly associated with lower psychopathology symptoms and higher well-being, whereas the opposite pattern was present for expressive suppression. Overall, the Polish version of the ERQ-S has strong psychometric properties and good clinical relevance.

## Graphical abstract

**Figure:**
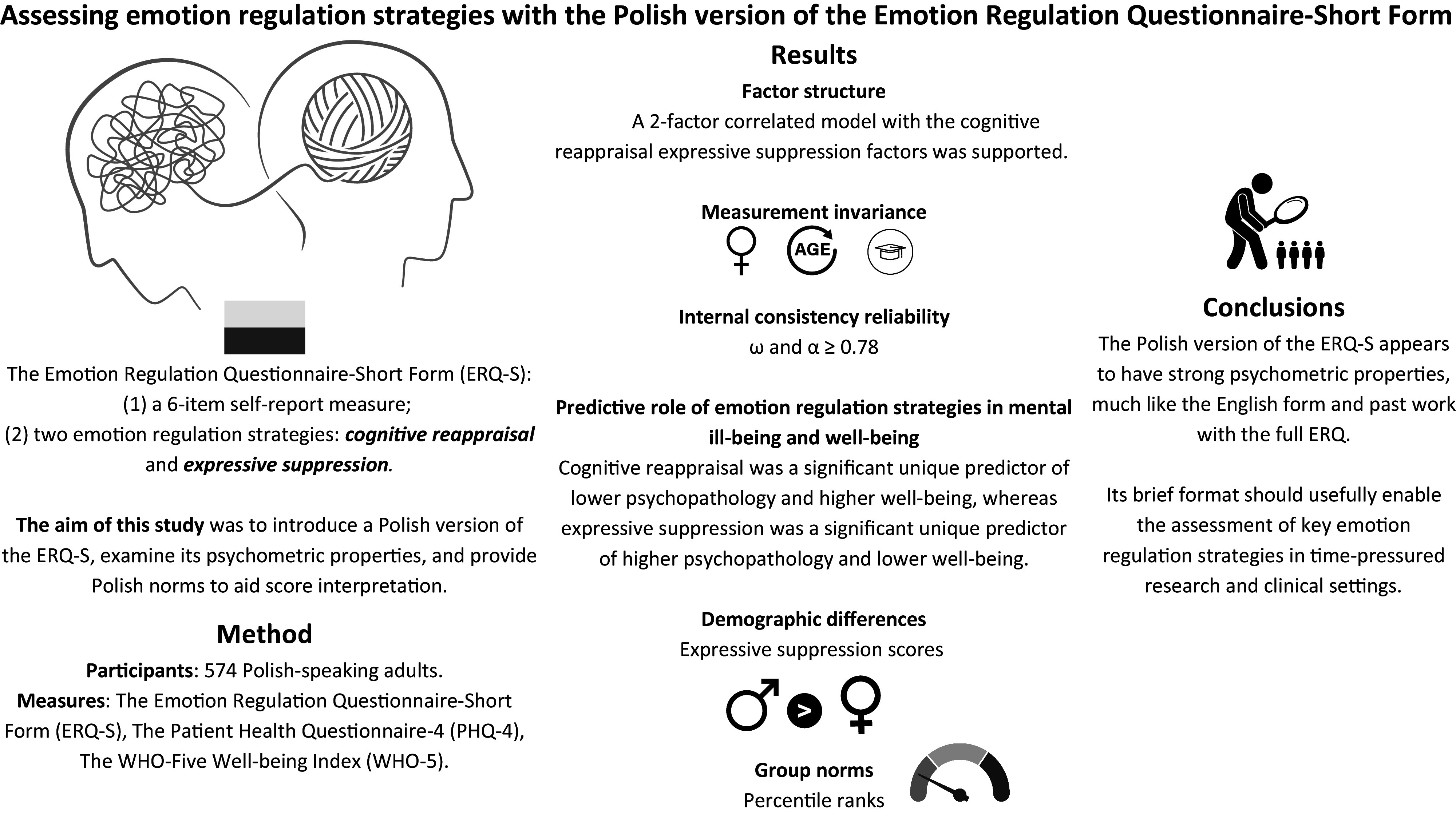

## Impact statement

Emotion regulation plays a crucial role in people’s emotional life. Therefore, its psychometric assessment is important in both research and clinical practice. Traditionally, most emotion regulation tools have been relatively lengthy, impacting their utility in time-pressured settings. Recently, the Emotion Regulation Questionnaire-Short Form (ERQ-S) was introduced in English as a brief measure to address this gap. In this study, we introduced the first Polish version of the ERQ-S and demonstrated that it has strong psychometric properties as a measure of two emotion regulation strategies, cognitive reappraisal and expressive suppression. The ERQ-S conformed well to its intended 2-factor structure in factor analysis and was invariant across gender, age and education categories. Moreover, the ERQ-S was able to maintain good reliability despite its brief format. We also highlighted that cognitive reappraisal was significantly associated with lower psychopathology symptoms and higher well-being, whereas the opposite pattern was present for expressive suppression. Overall, our findings further demonstrate the utility of the ERQ-S as a brief and robust measure of cognitive reappraisal and expressive suppression. To help facilitate interpretation of ERQ-S scores, we calculated percentile rank norms for Polish adults. The Polish version of the ERQ-S therefore can be recommended for use among Polish-speaking people around the world.

## Introduction

Emotion regulation plays a crucial role in people’s emotional life, with strong emotion regulation being associated with better overall well-being, and emotion regulation difficulties contributing to the development of a wide range of psychopathologies, including anxiety and depression disorders (Hu et al., 2014; Gratz et al., 2015; Brewer et al., 2016; Menefee et al., 2022).

A common model for conceptualizing emotion regulation is the process model of emotion regulation (Gross, 2015), which delineates five broad families of emotion regulation strategies, based on how early in the emotion generation process they are activated. Situation selection and situation modification strategies involve changing the emotion-inducing situations one encounters; attentional deployment strategies involve shifting what aspects of an emotion-inducing situation one focuses attention on; cognitive change strategies involve changing the way one is thinking about a situation to change its emotional impact; and response modulation strategies are activated later in the process once the emotional response is more developed, and involve modifying the experiential, physiological or behavioral manifestations of the emotion (Gross, 2015).

Assessing emotion regulation is of high importance in both research and clinical practice. For use in time-pressured research and clinical settings, brief and valid emotion regulation tools are required. To date, one of the most widely used measures of emotion regulation (See et al., 2022; Stellern et al., 2023; Zitzmann et al., 2024) has been the Emotion Regulation Questionnaire (ERQ) (Gross and John, 2003). The ERQ (Gross and John, 2003) is a 10-item self-report measure, which evaluates the extent of use of two common emotion regulation strategies: cognitive reappraisal and expressive suppression. Cognitive reappraisal is a cognitive change strategy involving reappraising the way one is thinking about a situation to change its emotional impact (e.g., looking at the situation from a different point of view), whereas expressive suppression is a response modulation strategy involving suppressing the behavioral expression of the emotion (e.g., trying to not show others how you are feeling; Gross and John, 2003).

These two emotion regulation strategies are of high clinical relevance (Gross and John, 2003). Cognitive reappraisal is generally considered an adaptive strategy, as its habitual use is associated with a wide range of positive outcomes, including lower psychopathology symptoms and better well-being and interpersonal functioning. In contrast, expressive suppression is generally considered a maladaptive strategy, as its habitual use is associated with poor long-term outcomes, such as higher psychopathology and lower well-being (Gross and John, 2003; Preece et al., 2020, 2021, 2023; Sörman et al., 2022).

Much of what is known about cognitive reappraisal and expressive suppression comes from work with the ERQ. The ERQ has shown good psychometric properties, including strong factorial validity with a theoretically informed 2-factor structure (i.e., cognitive reappraisal and expressive suppression factors) that works well across general community (e.g., Cabello et al., 2013; Preece et al., 2020; Olalde-Mathieu et al., 2022; Sörman et al., 2022), clinical (e.g., Andrea et al., 2023) and student (e.g., Balzarotti et al., 2010; Zhang and Bian, 2020) samples, and is invariant across various demographic groups (e.g., Ng et al., 2019; Preece et al., 2021). Both scale scores have displayed good internal consistency and test–retest reliabilities (e.g., Gómez-Ortiz et al., 2016; Olalde-Mathieu et al., 2022) and have been associated with other outcomes in expected directions. For example, ERQ cognitive reappraisal scores are robustly associated with better well-being and lower depression and anxiety, whereas the opposite pattern is present for ERQ expressive suppression scores (e.g., Preece et al., 2020, 2021; Sörman et al., 2022).

To further optimize the utility of the ERQ in time-pressured settings, Preece et al. (2023) recently introduced a 6-item short form called the Emotion Regulation Questionnaire-Short Form (ERQ-S). It is comprised of 3 cognitive reappraisal items and 3 expressive suppression items. To date, the psychometric properties of the ERQ-S have only been explored in one study (Preece et al., 2023), where the English version displayed strong performance in general community and college student samples from the United States, performing similarly to the full ERQ in terms of factor structure, internal consistency and relationships with a marker of depression and anxiety. Due to its short length, the ERQ-S might be a good option for assessing emotion regulation in clinical or research settings requiring brief assessments (e.g., busy clinical wards and studies where emotion regulation is assessed as a large battery of measures and there is a need for short measures to reduce participant fatigue). However, further work is needed to establish its psychometric performance. There is also a need for extensions into other cultures and language versions.

With this in mind, our aim in this study was to introduce the first Polish version of the ERQ-S and examine its psychometric properties in a Polish sample. We were also interested in providing general community norms for Polish adults to help facilitate the interpretation of ERQ-S scores. Based on the theory and past work on the ERQ-S and ERQ, we had several hypotheses (H).

H1. The intended 2-factor structure of the ERQ-S is a good fit to the data in confirmatory factor analysis.

H2. The ERQ-S’s factor structure demonstrates measurement invariance across gender, age and education categories.

H3. The ERQ-S has good internal consistency reliability.

H4. Higher levels of cognitive reappraisal correlate negatively with markers of anxiety and depression symptoms and positively with well-being, whereas higher levels of expressive suppression correlate positively with these psychopathology symptoms and negatively with well-being (refer to Preece et al., 2020; 2021; 2023; Sörman et al., 2022).

## Method

### Participants

Our sample consisted of 574 Polish-speaking adults (340 females, 227 males and 7 nonbinary) recruited from the general population in Poland, with ages ranging from 18 to 69 years (M = 25.47, SD = 9.03). Table 1 displays detailed demographic characteristics of the study sample, including education categories.

**Table 1.** Demographic characteristics of the study sample

Demographic characteristics			*n*	%
Age		*M* = 25.47, *SD* = 9.03, median = 22.00, min. = 18, max. = 69	574	100%
Gender		Females	340	59.23
		Males	227	39.55
		Nonbinary	7	1.22
Education	University degree (*n* = 184)	Higher	184	32.06
	No university degree (*n* = 390)	Secondary	339	59.06
		Vocational	27	4.70
		Primary	24	4.18

Abbreviations: M = mean; SD = standard deviation.

### Procedure

The study was conducted in accordance with the Declaration of Helsinki Ethical Principles. The Ethics Committee of the Faculty of Psychology of Kazimierz Wielki University approved the study (No. 1/13.06.2022, later revision November 28, 2023).

In this study, respondents were recruited using a purposeful sampling method with a maximum variation design (Palinkas et al., 2015). Participants were invited to complete a study in December 2023 via Facebook and Instagram, where we posted a link with an invitation to complete an online anonymous and voluntary survey (hosted on the Google Forms platform) with an appended consent form. No reimbursement was provided for the respondents. Participants provided their written informed consent digitally before completing the survey. Our inclusion criterion was Polish-speaking people aged 18 years or over, who signed their informed consent.

### Measures

***The Emotion Regulation Questionnaire-Short Form.*** The ERQ-S (Preece et al., 2023) is a 6-item self-report measure of two emotion regulation strategies: cognitive reappraisal and expressive suppression. Items are scored on a 7-point Likert scale ranging from 1 (“*strongly disagree*”) to 7 (“*strongly agree*”), with higher scores indicating higher usage of these strategies.

The Polish version of the ERQ-S was developed using a standard translation procedure (Wild et al., 2005). First, the original English version of the ERQ-S was translated into Polish by four independent translators. Based on their translations, a common Polish translation was developed. Second, we translated it back into English by an independent translator. This back translation was compared with the original version by one of the developers of the original ERQ-*S. Minor* corrections were made, resulting in the final Polish version of the ERQ-S (see Supplementary Materials for a copy of the Polish ERQ-S with its scoring instructions).

***The Patient Health Questionnaire-4 (PHQ-4).*** The PHQ-4 is a 4-item self-report measure of anxiety and depression symptoms over the previous two weeks (Kroenke et al., 2009). The PHQ-4 has two subscales: anxiety (e.g., “*Feeling nervous, anxious, or on edge*”) and depression (e.g., “*Feeling down, depressed, or hopeless*”), with two items in each subscale. The total PHQ-4 score represents an overall level of psychological distress. All PHQ-4 items are scored on a 4-point Likert scale, ranging from 0 (“*not at all*”) to 3 (“*nearly every day*”), with higher scores indicating higher levels of symptoms. We used the Polish version of the PHQ-4 (Larionow and Mudło-Głagolska, 2023).

***The WHO-Five Well-being Index (WHO-5).*** The WHO-5 is a 5-item self-report measure of positive well-being (WHO, 1998; Topp et al., 2015). Items (e.g., *“I feel cheerful and in good spirits*”) are scored on a 6-point Likert scale, ranging from 0 (*“at no time*”) to 5 (“*all the time*”), with higher scores indicating a higher level of well-being. We used the Polish version of the WHO-5 (Cichoń et al., 2020; Larionow, 2023).

The PHQ-4 and WHO-5 are widely used, and psychometrically sound brief measures of anxiety and depression symptoms (Caro-Fuentes and Sanabria-Mazo, 2023) or subjective well-being (Topp et al., 2015; Sischka et al., 2020), respectively. The Polish versions of these questionnaires have shown good psychometric properties (Cichoń et al., 2020; Larionow, 2023; Larionow and Mudło-Głagolska, 2023); therefore we felt these measures would be appropriate for assessing correlates of the Polish ERQ-S.

***The sociodemographic questionnaire***. All participants filled out a sociodemographic form on age, gender (females, males or nonbinary) as well as education degree. To avoid participants’ fatigue, the first part of the respondents (*n* = 302) completed all the measures (i.e., the ERQ-S, PHQ-4 and WHO-5), and the second part completed only the ERQ-S.

### Analytic strategy

Statistical analyses were carried out using *Statistica* v. 13.3 and *R* v. 4.3.0 with the *lavaan* statistical package. *JASP* v. 0.18.1 was used for calculating internal consistency reliability and an analysis of covariance (ANCOVA).

**Figure 1.**
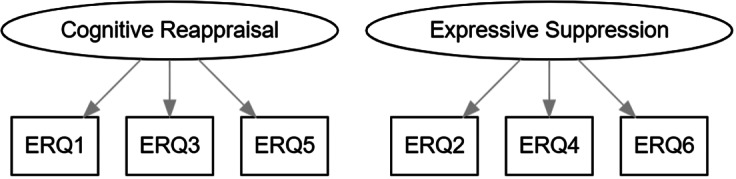
The tested confirmatory factor analysis model for the ERQ-S (the two factors were allowed to correlate).

***Factor structure and measurement invariance***. Confirmatory factor analysis with maximum likelihood estimation (robust standard errors and the Satorra–Bentler scaled test statistic) was used. We tested the theoretically informed 2-factor model of the ERQ-S, where items 1, 3 and 5 were specified to load on a “cognitive reappraisal” factor, and items 2, 4 and 6 on an “expressive suppression” factor (see Figure 1). The two factors were allowed to correlate.

To assess model goodness-of-fit, we used a variety of common fit indexes: the comparative fit index (CFI), the Tucker–Lewis index (TLI), root mean square error of approximation (RMSEA) and standardized root mean square residual (SRMR). CFI and TLI values ≥0.90 indicate acceptable fit and values ≥0.95 excellent fit. RMSEA and SRMR values ≤0.08 indicate acceptable fit and values ≤0.06 excellent fit (Hu and Bentler, 1999).

The measurement invariance of the factor structure was also examined across two gender categories (females vs. males), two age categories (younger people aged 18–24 vs. older people aged 25–69) and two education categories (no university degree vs. university degree; refer to Table 1). When dividing our sample into two age groups, we based the categorizations on the United Nations definition of youth, defining it as individuals between the ages of 15 and 24 (The United Nations Department of Economic and Social Affairs, 2013).

Configural, metric and scalar invariance models were tested. Models were compared in terms of the CFI, when an absolute difference in CFI (ΔCFI) of less than 0.01 supports invariance across the configural, metric and scalar levels (Cheung and Rensvold, 2002). Additionally, we applied criteria by Chen (2007), with ΔCFI of ≤0.01 for indicating invariance in all invariance models (i.e., metric and scalar), absolute differences in RMSEA of ≤0.015 and SRMR of ≤0.030 for indicating metric invariance, as well as with absolute differences in RMSEA of ≤0.015 and SRMR of ≤0.010 for indicating scalar invariance (Cieciuch and Davidov, 2015).

***Internal consistency reliability.*** McDonald’s omega (ω) and Cronbach’s alpha (α) reliability coefficients were calculated. Values ≥0.70 were judged as acceptable, ≥ 0.80 as good and ≥ 0.90 as excellent (Groth-Marnat, 2009).

***Concurrent validity.*** For assessing concurrent validity, we calculated Pearson correlations between ERQ-S scores, PHQ-4 scores (anxiety and depression symptoms) and WHO-5 scores (well-being).

***Predictive role of emotion regulation strategies in psychopathology and well-being**.* We conducted four separate multiple regression analyses in two steps using the forward entry method. In each regression, the criterion variable was either (1) anxiety symptoms (PHQ-4 anxiety), (2) depression symptoms (PHQ-4 depression), (3) total levels of psychopathology symptoms (PHQ-4 total score) or (4) well-being (WHO-5 total scores). In the first step of our regression models, we added age and gender as predictors to control demographic effects. In the second step, the two emotion regulation strategy scores of the ERQ-S were added as predictors.

***Demographic differences***. We computed Pearson correlations between ERQ-S scores and age in groups of females and males separately, as well as in the total sample. We used an ANCOVA with age as a covariate for comparing the ERQ-S scores between females and males, as well as between people with no university degree and people with a university degree. Age was used as a covariate in order to control its potential effects.

***Group norms***. We calculated percentile rank norms (Baumgartner, 2009) for the two ERQ-S scale scores in the total sample (*n* = 574).

## Results

### Descriptive statistics

Table 2 presents descriptive statistics for all study variables. In the total sample, across all the variables’ total or subscale scores, skewness ranged from −0.40 to 0.42, whereas kurtosis scores ranged from −1.13 to −0.53, indicating that the study variables were reasonably normally distributed. At the item level, all ERQ-S items were also reasonably normally distributed (see Supplementary Table 1).

**Table 2.** Descriptive statistics and internal consistency reliability coefficients for the study variables

Scale/subscale	Total sample					Females			Males			Nonbinary		
	*n*	ω (95% CI)	α (95% CI)	*M*	*SD*	*n*	*M*	*SD*	*n*	*M*	*SD*	*n*	*M*	*SD*
ERQ–S cognitive reappraisal	574	0.81 (0.77; 0.84)	0.81 (0.78; 0.83)	12.27	4.48	340	12.35	4.28	227	12.23	4.77	7	9.57	4.04
ERQ–S expressive suppression	574	0.78 (0.75; 0.81)	0.78 (0.75; 0.81)	13.62	4.76	340	13.18	4.75	227	14.29	4.67	7	13.71	6.05
PHQ–4 anxiety	302	0.73 (0.65; 0.78)	0.73 (0.66; 0.78)	3.33	1.77	257	3.33	1.78	40	3.10	1.60	5	5.40	0.89
PHQ–4 depression	302	0.81 (0.76; 0.85)	0.81 (0.76; 0.85)	2.67	1.90	257	2.67	1.91	40	2.45	1.75	5	4.60	2.07
PHQ–4 total score	302	0.85 (0.82; 0.88)	0.85 (0.82; 0.87)	6.00	3.37	257	5.99	3.41	40	5.55	2.91	5	10.00	2.12
WHO–5 total score	302	0.83 (0.79; 0.86)	0.83 (0.79; 0.86)	9.35	4.33	257	9.47	4.27	40	9.03	4.81	5	6.00	1.58

Abbreviations: ERQ-S = Emotion Regulation Questionnaire-Short Form; PHQ-4 = Patient Health Questionnaire-4; WHO-5 = WHO-Five Well-being Index; *M* = mean; *SD* = standard deviation, α = Cronbach’s alpha; ω = McDonald’s omega; 95% CI = 95% confidence interval.

### Factor structure and measurement invariance

In the total sample (*n* = 574), the intended 2-factor ERQ-S model was a good fit to the data (Table 3). Supplementary Table 1 displays standardized factor loadings of all ERQ-S items, which loaded well on their intended “cognitive reappraisal” and “expressive suppression” factors (loadings ≥0.683, all *ps* < 0.001). The estimated correlation between the cognitive reappraisal and expressive suppression factors was −0.139 (*p* = 0.008).

**Table 3.** Goodness-of-fit index values for the 2-factor model in the total sample, and measurement invariance across gender, age and education groups

Sample	χ^2^ (df)	CFI	TLI	RMSEA (90% CI)	SRMR	ΔCFI	ΔRMSEA	ΔSRMR	Invariance testing
Total sample (*n =* 574)	31.805 (8)	0.974	0.950	0.079 (0.051; 0.108)	0.053	–	–	–	–
Invariance models									
*Gender invariance*									
Configural	40.901 (16)	0.972	0.947	0.081 (0.051; 0.112)	0.051	–	–	–	–
Metric	45.740 (20)	0.971	0.957	0.073 (0.045; 0.101)	0.054	−0.001	−0.008	0.003	Supported
Scalar	47.519 (24)	0.974	0.968	0.063 (0.036; 0.089)	0.055	0.003	−0.010	0.001	Supported
*Age invariance*									
Configural	39.193 (16)	0.974	0.951	0.078 (0.047; 0.109)	0.047	–	–	–	–
Metric	41.291 (20)	0.976	0.965	0.066 (0.037; 0.095)	0.049	0.002	−0.012	0.002	Supported
Scalar	52.384 (24)	0.970	0.962	0.069 (0.043; 0.094)	0.051	−0.006	0.003	0.002	Supported
*Education invariance*									
Configural	50.945 (16)	0.962	0.929	0.095 (0.066; 0.125)	0.052	–	–	–	–
Metric	53.694 (20)	0.964	0.946	0.083 (0.056; 0.110)	0.053	0.002	−0.012	0.001	Supported
Scalar	61.318 (24)	0.961	0.952	0.078 (0.054; 0.103)	0.055	−0.003	−0.005	0.002	Supported

Abbreviations: χ^2^ = chi-square statistic; df = degrees of freedom; CFI = comparative fit index; TLI = Tucker–Lewis index; RMSEA = root mean square error of approximation; 90% CI = 90% confidence interval; SRMR = standardized root mean square residual.

We tested the configural, metric and scalar invariance of this 2-factor model across gender, age and education categories (Table 3). In all these analyses, the ΔCFI values were less than an absolute value of 0.01, indicating full metric and scalar invariance was supported for the ERQ-S across gender, age and education categories. Similarly, a more conservative measurement invariance analysis of ΔRMSEA and ΔSRMR strongly supported the full metric and scalar invariance across all the tested demographic categories.

### Internal consistency reliability

As demonstrated in Table 2, both ERQ-S scale scores had good internal consistency reliability (ω and α ≥ 0.78).

### Concurrent validity

Our correlational analyses (Supplementary Table 2) revealed that the ERQ-S cognitive reappraisal score was negatively correlated with psychopathology symptoms (*r* from −0.23 to −0.31, all *ps* < 0.001) and positively correlated with well-being (*r* = 0.37, *p* < 0.001), whereas the ERQ-S expressive suppression score was positively correlated with psychopathology symptoms (*r* from 0.24 to 0.26, all *ps* < 0.001) and negatively with well-being (*r* = −0.19, *p* < 0.001).

### Predictive role of emotion regulation strategies in mental ill-being and well-being

Our regression analyses indicated that the two emotion regulation strategies, as measured by the ERQ-S, were statistically significant predictors of psychopathology symptoms and well-being (see Table 4). At step 1, gender and age explained from 2.5% to 5% of the variance in psychopathology and well-being, whereas the two ERQ-S subscales explained a significant additional 6.6% to 13.0% of the variance. Across these regressions, cognitive reappraisal was a significant unique predictor of lower psychopathology and higher well-being, whereas expressive suppression was a significant unique predictor of higher psychopathology and lower well-being.

**Table 4.** Regression models for predicting mental ill-being and well-being (n = 302)

Predictors	Dependent variables							
	PHQ-4 anxiety		PHQ-4 depression		PHQ-4 total score		WHO-5 total score well-being	
	Step 1	Step 2	Step 1	Step 2	Step 1	Step 2	Step 1	Step 2
	Beta coefficients							
Gender	−0.05	−0.06	−0.05	−0.05	−0.06	−0.06	−0.03	−0.03
Age	−0.21***	−0.14*	−0.22***	−0.15**	−0.23***	−0.16**	0.17**	0.11
ERQ–S cognitive reappraisal	–	−0.19***	–	−0.27***	–	−0.25***	–	0.34***
ERQ–S expressive suppression	–	0.18**	–	0.17**	–	0.19***	–	−0.12*
Model parameters	*F*(2, 294) = 7.10, *p* < 0.001,	*F*(4, 292) = 9.77, *p* < 0.001	*F*(2, 294) = 7.56, *p* < 0.001	*F*(4, 292) = 13.35, *p* < 0.001	*F*(2, 294) = 8.76, *p* < 0.001	*F*(4, 292) = 13.92, *p* < 0.001	*F*(2, 294) = 4.79, *p* = 0.009	*F*(4, 292) = 14.62, *p* < 0.001
R^2^ adjusted	4.0%	10.6%	4.2%	14.3%	5.0%	14.9%	2.5%	15.5%
ΔR^2^ adjusted between the steps	6.6%		10.1%		9.9%		13.0%	

*Note:* *p < 0.05; **p < 0.01; ***p < 0.001. Gender was coded as following: females = 1; males = 2. Significant predictors are shown in bold.

Abbreviations: ERQ-S = Emotion Regulation Questionnaire-Short Form; PHQ-4 = Patient Health Questionnaire-4; WHO-5 = WHO-Five Well-being Index.

### Demographic differences

To explore demographic differences, we computed Pearson correlations between the two ERQ-S scale scores and age in the total sample and two gender groups separately (Supplementary Table 2). In females, age was positively correlated with the cognitive reappraisal subscale (*r* = 0.13, *p* < 0.05), whereas age was negatively correlated with the expressive suppression subscale (*r* = −0.21, *p* < 0.001). In males, there were no statistically significant correlations between age and the two ERQ-S strategies.

Two sets of ANCOVAs were used to compare ERQ-S scores across two gender (females vs. males) and two education categories (no university degree vs. university degree). Our results revealed no statistically significant gender differences in cognitive reappraisal scores, *F*(1, 564) = 0.01, *p* = 0.922. There were statistically significant gender differences in expressive suppression scores, *F*(1, 564) = 5.90, *p* = 0.015, η^2^ = 0.01; males reported using expressive suppression more often than females, though the effect size of this difference was small (η^2^ = 0.01). Cognitive reappraisal (*F*(1, 571) = 0.39, *p* = 0.533) and expressive suppression use (*F*(1, 571) = 0.29, *p* = 0.590) did not differ significantly across the education categories.

### Group norms

As gender differences were statistically significant only for the expressive suppression subscale with a small effect size, we calculated percentile rank norms for the ERQ-S in the total sample of Polish adults (see Supplementary Table 3). Based on the guide of using test scores of Flanagan and Caltabiano (2004), the percentile rank norms classification with low, average and high levels of characteristics was used. According to this guide, percentile ranks of ≤15 indicate low levels (with a label “low”), percentile ranks from 16 to 84 indicate average levels (with a label “average”) and percentile ranks of ≥85 indicate high levels (with a label “high”) of measured constructs. Our empirically derived percentile ranks were referred to using this percentile rank norms classification.

In our norms, percentile ranks of ≤15 indicate low levels of usage of the two emotion regulation strategies, percentile ranks from 16 to 84 indicate average levels and percentile ranks of ≥85 indicate high levels. For example, if the participant has an ERQ-S cognitive reappraisal score of 11, and this refers to a percentile rank of 36, all that means that this participant tends to use the cognitive reappraisal strategy more often than 36% of people in a reference group. In order to simplify the interpretation of these norms, we present labels “low,” “average” and “high” for percentile ranks for each ERQ-S strategy (Supplementary Table 3).

## Discussion

In this study, we introduced the Polish version of the ERQ-S and examined its psychometric properties. Overall, the validity and reliability of this questionnaire were supported, thus supporting that the ERQ-S seems to be a strong option for the brief assessment of key emotion regulation strategies.

***Psychometric properties.*** We found good support for the intended 2-factor structure of the ERQ-S, corresponding to the two targeted emotion regulation strategies (i.e., cognitive reappraisal and expressive suppression). These results are in line with the original validation study of the ERQ-S (Preece et al., 2023), as well as the large body of previous factor analytic work on the full ERQ (e.g., Balzarotti et al., 2010; Cabello et al., 2013; Ng et al., 2019; Preece et al., 2020, 2021; Sörman et al., 2022). Moreover, we also tested the invariance of the ERQ-S across different gender, age and education categories and found that the ERQ-S was invariant across these different demographic groups. Thus, the ERQ-S appears to perform similarly across (1) females and males, (2) younger and older people as well as (3) people with a university degree and those without a university degree. This means that the ERQ-S can meaningfully assess differences in emotion regulation strategy use across these demographic groups.

Despite the brevity of the two 3-item ERQ-S scales, the Polish version of the ERQ-S showed good internal consistency reliability, reaching thresholds desired for use in both research and clinical settings. The two ERQ-S scale scores also correlated in expected directions with psychopathology symptoms and well-being, explaining significant variance in these clinically relevant outcomes, which is in line with results from the previous ERQ-S study (Preece et al., 2023) as well as work with the full ERQ (e.g., Balzarotti et al., 2010; Cabello et al., 2013; Preece et al., 2020, 2021; Sörman et al., 2022). Cognitive reappraisal, as assessed by the ERQ-S, therefore appears to be a broadly adaptive strategy, whereas expressive suppression appears to be a broadly maladaptive strategy (Hu et al., 2014). In our study, the links between the ERQ-S subscales and the examined correlates (i.e., psychopathology symptoms and well-being) were small to moderate, supporting the results of previous meta-analytic studies where other emotion regulation measures, including the full ERQ, were used (Aldao et al., 2010; Kraiss et al., 2020). Overall, the ERQ-S therefore appears to maintain the good clinical relevance of the full form (Gross and John, 2003).

***Demographic comparisons***. Controlling for age, our results revealed no statistically significant gender differences in cognitive reappraisal scores on the ERQ-S, whereas there were statistically significant gender differences in expressive suppression scores (males reported higher scores than females) with the small effect size, which is in line with the past works (e.g., Zhang and Bian, 2020; Olalde-Mathieu et al., 2022). Controlling for age, no statistically significant education differences in ERQ-S scores were noted, supporting the conclusions presented in previous reports (Nakagawa et al., 2017).

As for age differences in habitual use of the two emotion regulation strategies, past research with the ERQ had shown a mix of findings. For example, positive relationships between age and these two strategies (e.g., Nakagawa et al., 2017; García et al., 2023) or a negative link with expressive suppression (Olalde-Mathieu et al., 2022) were indicated in some studies. In others, no significant differences were found between younger and older people in expressive suppression scores, but significantly higher cognitive reappraisal scores in younger people were indicated (Oriyama et al., 2024). Our previous studies have shown that associations with age and emotional variables can differ across gender categories (Larionow et al., 2022, 2023a, 2023b; Larionow and Mudło-Głagolska, 2022). Therefore, we assessed links between age and ERQ-S scores in the total sample, and in females and males separately.

Our correlational analysis indicated that older females tended to have more favorable emotion regulation (i.e., higher levels of cognitive reappraisal and lower levels of expressive suppression), whereas males tended to have relatively stable levels of emotion regulation strategy use regardless of age. This conclusion is limited, because our study was cross-sectional and not longitudinal. Notwithstanding, these findings are in line with the previous Polish studies on age–gender correlational patterns within psychosomatic variables (e.g., alexithymia, emotional reactivity and somatic complaints), which indicated a shift to a more favorable emotional functioning in females with age (but not in males; e.g., Larionow and Mudło-Głagolska, 2022; Larionow et al., 2022, 2023a, 2023b).

***Group norms and their interpretations***. To help facilitate interpretation of ERQ-S scores, we calculated percentile rank norms for our total sample of Polish adults. Our data indicate that in a Polish context, a score of ≤7 on the ERQ-S cognitive reappraisal scale is an indicator of “low usage” of this strategy, whereas a score of ≥19 in expressive suppression is an indicator of “high usage” of this strategy. An individual with low cognitive reappraisal scores and/or with high expressive suppression scores is potentially in a high-risk group for mental disorders, with poorer emotion regulation skills (Cutuli, 2014).

The norms provided here may therefore help to identify people in need of emotion regulation-focused interventions and therefore guide the targeting of psychological interventions. The ERQ-S might be used for assessing emotion regulation strategies before and after treatment, or as part of clinical trials. Being a short measure, the ERQ-S can be also administered in general population studies for screening assessments of emotion regulation. Longitudinal and cross-cultural studies may benefit from the use of the ERQ-S due to its brevity and the cross-cultural applicability of the emotion regulation construct.

***Limitations of the study and future directions***. This was a study based on self-report measures, which can have several disadvantages, including vulnerabilities to social desirability and response biases (Demetriou et al., 2015). Surveys like ours, which are anonymous and where participation does not have reimbursement, can help reduce some of these concerns, though it still remains an important consideration. Based on our cross-sectional study, no conclusions can be drawn regarding the cause-and-effect relationship of emotion regulation strategy use and other study variables. The test–retest reliability of the Polish ERQ-S was not examined in this study.

The psychometric properties of the questionnaire were assessed in a general community sample, without testing in clinical samples, so future work in clinical samples will be important. Therefore, future research will be required to test the generalizability of our findings in different samples and settings. To help reveal possible psychological mechanisms underlying the development of positive and negative mental health outcomes, future studies focused on ERQ-S emotion regulation profiles (i.e., a combination of various emotion regulation strategies), and their relationships with markers of ill-being and well-being, will be useful.

Being a short measure, the ERQ-S can be used in clinical wards or large-scale epidemiological studies where many questionnaires of different constructs may need to be administered, thus requiring each measure to have a minimally optimal item set. There can also be applications in ecological momentary assessment research designs, where questionnaires often need to be administered multiple times a day over several days, thus necessitating short formats (Koval et al., 2020). Use of short measures of emotion regulation, like the ERQ-S, can enable the inclusion of more measures of other constructs while accounting for participant fatigue, hence enabling more complex studies or research designs (Preece et al., 2023).

## Conclusions

The Polish version of the ERQ-S appears to have strong psychometric properties, much like the English form (Preece et al., 2023) and past work with the full ERQ (Gross and John, 2003). Its brief format should usefully enable the assessment of key emotion regulation strategies in time-pressured research and clinical settings.

## Supporting information

Larionow et al. supplementary material Larionow et al. supplementary material

## Data Availability

The data that support the findings of this study are available from the corresponding author, P.L., upon reasonable request.

## Supplementary material

The supplementary material for this article can be found at http://doi.org/10.1017/gmh.2024.97.

## References

[r1] Aldao A, Nolen-Hoeksema S and Schweizer S (2010) Emotion-regulation strategies across psychopathology: A meta-analytic review. Clinical Psychology Review 30(2), 217–237. 10.1016/j.cpr.2009.11.004. 20015584

[r2] Andrea AM, Galiano CS, Rosellini AJ and Brown TA (2023) Psychometric evaluation of the Emotion Regulation Questionnaire in a clinical sample. Journal of Psychopathology and Behavioral Assessment 46, 1–9. 10.1007/s10862-023-10108-x. PMC12846327 41607397

[r3] Balzarotti S, John OP and Gross JJ (2010) An Italian adaptation of the emotion regulation questionnaire. European Journal of Psychological Assessment 26(1), 61–67. 10.1027/1015-5759/a000009.

[r4] Baumgartner TA (2009) Tutorial: Calculating percentile rank and percentile norms using SPSS. Measurement in Physical Education and Exercise Science 13(4), 227–233. 10.1080/10913670903262769.

[r5] Brewer SK, Zahniser E and Conley CS (2016) Longitudinal impacts of emotion regulation on emerging adults: Variable- and person-centered approaches. Journal of Applied Developmental Psychology 47, 1–12. 10.1016/j.appdev.2016.09.002.

[r6] Cabello R, Salguero JM, Fernández-Berrocal P and Gross JJ (2013) A Spanish adaptation of the emotion regulation questionnaire. European Journal of Psychological Assessment 29(4), 234–240. 10.1027/1015-5759/a000150.

[r7] Caro-Fuentes S and Sanabria-Mazo JP (2023) A systematic review of the psychometric properties of the Patient Health Questionnaire-4 in clinical and nonclinical populations. Journal of the Academy of Consultation-Liaison Psychiatry 65, 178–194. 10.1016/j.jaclp.2023.11.685. 38012988

[r8] Chen FF (2007) Sensitivity of goodness of fit indexes to lack of measurement invariance. Structural Equation Modeling: A Multidisciplinary Journal 14(3), 464–504. 10.1080/10705510701301834.

[r9] Cheung GW and Rensvold RB (2002) Evaluating goodness-of-fit indexes for testing measurement invariance. Structural Equation Modeling: A Multidisciplinary Journal 9(2), 233–255. 10.1207/S15328007SEM0902_5.

[r10] Cichoń E, Kiejna A, Kokoszka A, Gondek T, Rajba B, Lloyd CE and Sartorius N (2020) Validation of the polish version of WHO-5 as a screening instrument for depression in adults with diabetes. Diabetes Research and Clinical Practice 159, 107970. 10.1016/j.diabres.2019.107970. 31805355

[r11] Cieciuch J and Davidov E (2015) Establishing measurement invariance across online and offline samples. A tutorial with the software packages Amos and Mplus. Studia Psychologica: Theoria Et Praxis 15(2), 83–99. 10.21697/sp.2015.14.2.06.

[r12] Cutuli D (2014) Cognitive reappraisal and expressive suppression strategies role in the emotion regulation: An overview on their modulatory effects and neural correlates. Frontiers in Systems Neuroscience 8, 175. 10.3389/fnsys.2014.00175. 25285072 PMC4168764

[r13] Demetriou C, Ozer BU and Essau CA (2015) Self-report questionnaires. In Cautin RL and Lilienfeld SO (eds.), The Encyclopedia of Clinical Psychology. John Wiley & Sons, Inc, pp. 1–6. 10.1002/9781118625392.wbecp507.

[r14] Flanagan DP and Caltabiano LF (2004) Test scores: A guide to understanding and using test results. In Canter AS, Paige LZ, Roth MD, Romero I, Carroll SA (eds.), Helping Children at Home and School II: Handouts for Families and Educators. Bethesda: National Association of School Psychologists, pp. 81–84.

[r15] García FE, Vergara-Barra P, Concha-Ponce P, Andrades M, Rincón P and Valdivia-Devia M (2023) The emotion regulation questionnaire: Psychometric properties and prediction of posttraumatic consequences during the COVID-19 pandemic in Chilean adults. International Journal of Environmental Research and Public Health 20(4), 3452. 10.3390/ijerph20043452. 36834148 PMC9967314

[r16] Gómez-Ortiz O, Romera EM, Ortega-Ruiz R, Cabello R and Fernández-Berrocal P (2016) Analysis of emotion regulation in Spanish adolescents: Validation of the emotion regulation questionnaire. Frontiers in Psychology 6, 1959. 10.3389/fpsyg.2015.01959. 26779076 PMC4703776

[r17] Gratz KL, Weiss NH and Tull MT (2015) Examining emotion regulation as an outcome, mechanism, or target of psychological treatments. Current Opinion in Psychology 3, 85–90. 10.1016/j.copsyc.2015.02.010. 25859561 PMC4386282

[r18] Gross JJ (2015) Emotion regulation: Current status and future prospects. Psychological Inquiry 26(1), 1–26. 10.1080/1047840X.2014.940781.

[r19] Gross JJ and John OP (2003) Individual differences in two emotion regulation processes: Implications for affect, relationships, and well-being. Journal of Personality and Social Psychology 85(2), 348–362. 10.1037/0022-3514.85.2.348. 12916575

[r20] Groth-Marnat G (2009) Handbook of Psychological Assessment, 5th Edn. Hoboken, New Jersey: Wiley.

[r21] Hu LT and Bentler PM (1999) Cutoff criteria for fit indexes in covariance structure analysis: Conventional criteria versus new alternatives. Structural Equation Modeling: A Multidisciplinary Journal 6, 1–55. 10.1080/10705519909540118.

[r22] Hu T, Zhang D, Wang J, Mistry R, Ran G and Wang X (2014) Relation between emotion regulation and mental health: A meta-analysis review. Psychological Reports 114(2), 341–362. 10.2466/03.20.PR0.114k22w4. 24897894

[r23] Koval P, Kalokerinos EK, Verduyn P and Greiff S (2020) Introduction to the special issue: Capturing the dynamics of emotion and emotion regulation in daily life with ambulatory assessment. European Journal of Psychological Assessment, 36(3), 433–436. 10.1027/1015-5759/a000599.

[r24] Kraiss JT, ten Klooster PM, Moskowitz JT and Bohlmeijer ET (2020) The relationship between emotion regulation and well-being in patients with mental disorders: A meta-analysis. Comprehensive Psychiatry 102, 152189. 10.1016/j.comppsych.2020.152189. 32629064

[r25] Kroenke K, Spitzer RL, Williams JB and Löwe B (2009) An ultra-brief screening scale for anxiety and depression: The PHQ-4. Psychosomatics 50(6), 613–621. 10.1176/appi.psy.50.6.613. 19996233

[r26] Larionow P (2023) Anxiety and depression screening among Polish adults in 2023: Depression levels are higher than in cancer patients. Psychiatria 20, 143–151. 10.5603/psych.97199.

[r27] Larionow P and Mudło-Głagolska K (2022) Assessment of activation, intensity and duration of positive and negative emotions: Psychometric properties of the Polish version of the Perth Emotional Reactivity Scale – Short Form. Current Issues in Personality Psychology 12, 60–72. 10.5114/cipp/156146. 38756198 PMC11094455

[r28] Larionow P and Mudło-Głagolska K (2023) The patient health Questionnaire-4: Factor structure, measurement invariance, latent profile analysis of anxiety and depressive symptoms and screening results in polish adults. Advances in Cognitive Psychology 19(2), 123–137. 10.5709/acp-0391-2.

[r29] Larionow P, Mudło-Głagolska K and Michalak M (2022) Towards psychosomatic medicine: Psychometric properties of the polish version of the Giessen subjective complaints list (GBB-8) and the prevalence of somatic symptoms in a polish community sample. Annales Universitatis Mariae Curie-Skłodowska, sectio J – Paedagogia-Psychologia 35(4), 117–138. 10.17951/j.2022.35.4.117-138.

[r30] Larionow P, Preece DA and Mudło-Głagolska K (2023a) Psychometric properties of the polish version of the Perth emotional reactivity scale. International Journal of Cognitive Therapy 16, 460–478. 10.1007/s41811-023-00172-2.

[r31] Larionow P, Preece DA and Mudło-Głagolska K (2023b) The polish version of the Perth alexithymia questionnaire-short form (PAQ-S): Psychometric properties and norms. Journal of Sexual and Mental Health 21, 54–63. 10.5603/jsmh.97351.

[r32] Menefee DS, Ledoux T and Johnston CA (2022) The importance of emotional regulation in mental health. American Journal of Lifestyle Medicine 16(1), 28–31. 10.1177/15598276211049771. 35185423 PMC8848120

[r33] Nakagawa T, Gondo Y, Ishioka Y and Masui Y (2017) Age, emotion regulation, and affect in adulthood: The mediating role of cognitive reappraisal. Japanese Psychological Research 59, 301–308. 10.1111/jpr.12159.

[r34] Ng ZJ, Huebner ES, Maydeu-Olivares A and Hills KJ (2019) Confirmatory factor analytic structure and measurement invariance of the emotion regulation questionnaire for children and adolescents in a longitudinal sample of adolescents. Journal of Psychoeducational Assessment 37(2), 139–153. 10.1177/0734282917732891.

[r35] Olalde-Mathieu VE, Licea-Haquet G, Reyes-Aguilar A and Barrios FA (2022) Psychometric properties of the emotion regulation questionnaire in a Mexican sample and their correlation with empathy and alexithymia. Cogent Psychology 9(1), 2053385. 10.1080/23311908.2022.2053385.

[r36] Oriyama K, Mukai K, Harada K and Masumoto K (2024) Relationship between habitual use and degree of emotion regulation: Age differences in cognitive reappraisal and expressive suppression. Experimental Aging Research, 1–14. 10.1080/0361073X.2024.2315917. 38372075

[r37] Palinkas LA, Horwitz SM, Green CA, Wisdom JP, Duan N and Hoagwood K (2015) Purposeful sampling for qualitative data collection and analysis in mixed method implementation research. Administration and Policy in Mental Health 42(5), 533–544. 10.1007/s10488-013-0528-y. 24193818 PMC4012002

[r38] Preece DA, Becerra R, Hasking P, McEvoy PM, Boyes M, Sauer-Zavala S, Chen W and Gross JJ (2021) The emotion regulation questionnaire: Psychometric properties and relations with affective symptoms in a United States general community sample. Journal of Affective Disorders 284, 27–30. 10.1016/j.jad.2021.01.071. 33582429

[r39] Preece DA, Becerra R, Robinson K and Gross JJ (2020) The emotion regulation questionnaire: Psychometric properties in general community samples. Journal of Personality Assessment 102(3), 348–356. 10.1080/00223891.2018.1564319. 30714818

[r40] Preece DA, Petrova K, Mehta A and Gross JJ (2023) The emotion regulation questionnaire-short form (ERQ-S): A 6-item measure of cognitive reappraisal and expressive suppression. Journal of Affective Disorders 340, 855–861. 10.1016/j.jad.2023.08.076. 37597776

[r41] See CCH, Tan JM, Tan VSY and Sündermann O (2022) A systematic review on the links between emotion regulation difficulties and obsessive-compulsive disorder. Journal of Psychiatric Research 154, 341–353. 10.1016/j.jpsychires.2022.07.023. 36049435

[r42] Sischka PE, Costa AP, Steffgen G and Schmidt AF (2020) The WHO-5 well-being index – Validation based on item response theory and the analysis of measurement invariance across 35 countries. Journal of Affective Disorders Reports 1, 100020. 10.1016/j.jadr.2020.100020.

[r43] Sörman K, Garke MÅ, Isacsson NH, Jangard S, Bjureberg J, Hellner C, Sinha R and Jayaram-Lindström N (2022) Measures of emotion regulation: Convergence and psychometric properties of the difficulties in emotion regulation scale and emotion regulation questionnaire. Journal of Clinical Psychology 78(2), 201–217. 10.1002/jclp.23206. 34217149

[r44] Stellern J, Xiao KB, Grennell E, Sanches M, Gowin JL and Sloan ME (2023) Emotion regulation in substance use disorders: A systematic review and meta-analysis. Addiction 118(1), 30–47. 10.1111/add.16001. 35851975 PMC10087816

[r45] The United Nations Department of Economic and Social Affairs (2013). Definition of youth. Available at https://www.un.org/esa/socdev/documents/youth/fact-sheets/youth-definition.pdf.

[r46] Topp CW, Østergaard SD, Søndergaard S and Bech P (2015) The WHO-5 well-being index: A systematic review of the literature. Psychotherapy and Psychosomatics 84(3), 167–176. 10.1159/000376585. 25831962

[r47] Wild D, Grove A, Martin M, Eremenco S, McElroy S, Verjee-Lorenz A and Erikson P (2005) Principles of good practice for the translation and cultural adaptation process for patient-reported outcomes (PRO) measures: Report of the ISPOR task force for translation and cultural adaptation. Value in Health 8(2), 94–104. 10.1111/j.1524-4733.2005.04054.x. 15804318

[r48] World Health Organization. Regional Office for Europe (1998) Wellbeing Measures in Primary Health Care/the DepCare Project: Report on a WHO Meeting: Stockholm, Sweden, 12–13 February 1998. Copenhagen: World Health Organization. Regional Office for Europe. https://apps.who.int/iris/handle/10665/349766.

[r49] Zhang Y and Bian Y (2020) Emotion regulation questionnaire for cross-gender measurement invariance in Chinese university students. Frontiers in Psychology 11, 569438. 10.3389/fpsyg.2020.569438. 33250813 PMC7673430

[r50] Zitzmann J, Rombold-George L, Rosenbach C and Renneberg B (2024) Emotion regulation, parenting, and psychopathology: A systematic review. Clinical Child and Family Psychology Review 27(1), 1–22. 10.1007/s10567-023-00452-5. PMC10920465 37704867

