# Supplementary material for: The Polish version of the Emotion Regulation Questionnaire-Short Form (ERQ-S): Psychometric properties, Polish norms and relationships with psychopathology and well-being
Source: Glob Ment Health (Camb). 2024 Oct 28;11:e104. doi: 10.1017/gmh.2024.97 (PMC11518626; doi:10.1017/gmh.2024.97)
Supplement: Larionow et al. supplementary material [file S2054425124000979sup001.docx]

**Supplementary Table 1**

*Descriptive statistics of the ERQ-S statements and standardized factor loadings from confirmatory factor analysis (n = 574)*

| ERQ-S statements | *M* | *SD* | Skewness | Kurtosis | Factor loadings |
| --- | --- | --- | --- | --- | --- |
| 1. “When I want to feel more *positive* emotion (such as joy or amusement), I *change the way I’m thinking* about the situation.” | 4.23 | 1.75 | -0.21 | -0.86 | 0.714 |
| 2. “I keep my emotions to myself.” | 4.83 | 1.85 | -0.57 | -0.86 | 0.724 |
| 3. “When I want to feel less *negative* emotion (such as sadness or anger), I *change the way I’m thinking* about the situation.” | 4.09 | 1.83 | -0.13 | -0.98 | 0.818 |
| 4. “I control my emotions *by not expressing them*.” | 4.29 | 1.95 | -0.26 | -1.14 | 0.806 |
| 5. “I control my emotions by *changing the way I think* about the situation I’m in.” | 3.95 | 1.70 | -0.04 | -0.87 | 0.757 |
| 6. “When I am feeling *negative* emotions, I make sure not to express them.” | 4.50 | 1.91 | -0.33 | -1.09 | 0.683 |

*Note*. ERQ-S = Emotion Regulation Questionnaire-Short Form; *M* = mean; *SD* = standard deviation. All factor loadings are statistically significant (*ps* < 0.001).

**Supplementary Table 2**

*Pearson correlations between the ERQ-S and age, PHQ-4, and WHO-5 scores*

| Variables | ERQ-S Cognitive reappraisal | ERQ-S Expressive suppression |
| --- | --- | --- |
| Age (females, *n* = 340) | 0.13* | -0.21*** |
| Age (males, *n* = 227) | 0.04 | -0.06 |
| Age (total sample, *n* = 574) | 0.10* | -0.16*** |
| PHQ-4 Anxiety (*n* = 302) | -0.23*** | 0.24*** |
| PHQ-4 Depression (*n* = 302) | -0.31*** | 0.24*** |
| PHQ-4 Total score (*n* = 302) | -0.29*** | 0.26*** |
| WHO-5 Total score (*n* = 302) | 0.37*** | -0.19*** |

*Note*. * = *p* < 0.05; *** = *p* < 0.001.

**Supplementary Table 3**

*Percentile rank norms for the ERQ-S in the total sample of Polish adults (n = 574)*

| ERQ-S Cognitive reappraisal | | | ERQ-S Expressive suppression | | |
| --- | --- | --- | --- | --- | --- |
| Raw score | Percentile rank | Norms interpretations | Raw score | Percentile rank | Norms interpretations |
| 3 | 2.3 | Low | 3 | 1.4 | Low |
| 4 | 5.0 |  | 4 | 3.3 |  |
| 5 | 7 |  | 5 | 5.0 |  |
| 6 | 11 |  | 6 | 8 |  |
| 7 | 15 |  | 7 | 11 |  |
| 8 | 18 | Average | 8 | 15 |  |
| 9 | 24 |  | 9 | 20 | Average |
| 10 | 30 |  | 10 | 26 |  |
| 11 | 36 |  | 11 | 29 |  |
| 12 | 45 |  | 12 | 34 |  |
| 13 | 54 |  | 13 | 41 |  |
| 14 | 62 |  | 14 | 48 |  |
| 15 | 72 |  | 15 | 56 |  |
| 16 | 80 |  | 16 | 63 |  |
| 17 | 85 | High | 17 | 72 |  |
| 18 | 90 |  | 18 | 81 |  |
| 19 | 94 |  | 19 | 87 | High |
| 20 | 95.7 |  | 20 | 92 |  |
| 21 | 98.1 |  | 21 | 97.0 |  |

*Note*. Percentile ranks of ≤5 and ≥95 are reported with one decimal place, and percentile ranks of >5 and <95 are reported as integers.

**Supplementary Materials**

*The Polish version of the Emotion Regulation Questionnaire-Short Form (ERQ-S)*

**Kwestionariusz regulacji emocji – Krótka wersja**

**Instrukcja i twierdzenia**

Chcielibyśmy zadać Ci kilka pytań dotyczących Twojego życia emocjonalnego, w szczególności tego, jak kontrolujesz emocje (to znaczy jak je regulujesz lub jak nimi zarządzasz). Poniższe pytania obejmują dwa aspekty Twojego życia emocjonalnego. Pierwszy aspekt to Twoje doświadczenia emocjonalne, czyli to, co czujesz w środku. Drugi aspekt dotyczy Twojego wyrażania emocji, czyli tego, jak pokazujesz swoje emocje w sposobie mówienia, gestykulacji i zachowania. Chociaż niektóre z poniższych pytań mogą wydawać się podobne do siebie, różnią się one w istotny sposób. Odpowiedz, proszę, na każde pytanie, wpisując przy każdym twierdzeniu odpowiednią cyfrę z następującej skali:

| **1** | | | **2** | **3** | **4** | **5** | **6** | **7** |
| --- | --- | --- | --- | --- | --- | --- | --- | --- |
| **zdecydowanie**  **się nie zgadzam** | | | **---------------------** | **---------------------** | **ani się zgadzam, ani się nie zgadzam** | **---------------------** | **---------------------** | **zdecydowanie się zgadzam** |
| 1 | ____ | Kiedy chcę poczuć więcej *pozytywnych* emocji (takich jak radość lub rozbawienie), *zmieniam swój sposób myślenia* o danej sytuacji. | | | | | | |
| 2 | ____ | Trzymam swoje emocje w sobie. | | | | | | |
| 3 | ____ | Kiedy chcę poczuć mniej *negatywnych* emocji (takich jak smutek lub złość), *zmieniam swój sposób myślenia* o danej sytuacji. | | | | | | |
| 4 | ____ | Kontroluję swoje emocje poprzez *niewyrażanie/nieokazywanie ich*. | | | | | | |
| 5 | ____ | Kontroluję swoje emocje, *zmieniając swój sposób myślenia* o sytuacji, w której się znajduję. | | | | | | |
| 6 | ____ | Kiedy odczuwam *negatywne* emocje, staram się ich nie okazywać. | | | | | | |

**Instrukcja obliczania wyników**

*Kwestionariusz regulacji emocji – Krótka wersja* (Emotion Regulation Questionnaire – Short Form; ERQ-S; Preece i in., 2023) to 6-pozycyjna skrócona wersja *Kwestionariusza regulacji emocji* (Emotion Regulation Questionnaire; ERQ; Gross i John, 2003).

Podobnie jak ERQ, ERQ–S służy do oceny różnic indywidualnych w nawykowym stosowaniu dwóch strategii regulacji emocji: *przeformułowania poznawczego* (tj. zmiany sposobu myślenia o sytuacji w celu zmiany jej wpływu emocjonalnego) i *tłumienia ekspresji* (tj. tłumienia behawioralnej ekspresji emocji). Wyniki ERQ–S oblicza się oddzielnie dla każdej z tych strategii, przy czym wyższe wyniki wskazują na wyższy poziom wykorzystywania tych strategii.

Wysoki poziom korzystania z przeformułowania poznawczego jest zwykle związany z dobrym samopoczuciem i wysoką jakością relacji interpersonalnych, podczas gdy wysoki poziom korzystania z tłumienia ekspresji jest zwykle związany z obniżonym samopoczuciem i niską jakością relacji interpersonalnych (Gross i John, 2003). ERQ–S charakteryzował się dobrymi właściwościami psychometrycznymi, podobnie jak pełna wersja ERQ (Preece i in., 2023).

**Adnotacja**

Nie zmieniaj kolejności pozycji, ponieważ pozycje 1 i 3 na początku kwestionariusza definiują terminy „pozytywne emocje” i „negatywne emocje”.

**Obliczanie wyników (brak pozycji odwróconych)**

Przeformułowanie poznawcze: zsumuj wyniki pozycji 1, 3, 5.

Tłumienie ekspresji: zsumuj wyniki pozycji 2, 4, 6.
