# Supplementary material for: The Polish version of the Emotion Regulation Questionnaire-Short Form (ERQ-S): Psychometric properties, Polish norms and relationships with psychopathology and well-being
Source: Glob Ment Health (Camb). 2024 Oct 28;11:e104. doi: 10.1017/gmh.2024.97 (PMC11518626; doi:10.1017/gmh.2024.97)
Supplement: Peer review history

# Editor report: Recommendation: The Polish version of the Emotion Regulation Questionnaire-Short Form (ERQ-S): Psychometric properties, Polish norms and relationships with psychopathology and well-being — R0/PR2

**DOI:** 10.1017/gmh.2024.97.pr2

**Affiliations:** Primary Healthcare Sciences, University of Zimbabwe Faculty of Medicine, Zimbabwe

Kindly address the methodological issues raised by the reviewers, particularly the translation procedure and participant recruitment and clearly outline the data analysis.

# Editor report: Decision: The Polish version of the Emotion Regulation Questionnaire-Short Form (ERQ-S): Psychometric properties, Polish norms and relationships with psychopathology and well-being — R0/PR3

**DOI:** 10.1017/gmh.2024.97.pr3

**Affiliations:** Johns Hopkins University Bloomberg School of Public Health, United States

No accompanying comment.

# Editor report: Recommendation: The Polish version of the Emotion Regulation Questionnaire-Short Form (ERQ-S): Psychometric properties, Polish norms and relationships with psychopathology and well-being — R1/PR5

**DOI:** 10.1017/gmh.2024.97.pr5

**Affiliations:** Primary Healthcare Sciences, University of Zimbabwe Faculty of Medicine, Zimbabwe

No accompanying comment.

# Editor report: Decision: The Polish version of the Emotion Regulation Questionnaire-Short Form (ERQ-S): Psychometric properties, Polish norms and relationships with psychopathology and well-being — R1/PR6

**DOI:** 10.1017/gmh.2024.97.pr6

**Affiliations:** Johns Hopkins University Bloomberg School of Public Health, United States

No accompanying comment.
